# 血清碳酸酐酶Ⅸ检测对肺癌的诊断意义

**DOI:** 10.3779/j.issn.1009-3419.2015.01.05

**Published:** 2015-01-20

**Authors:** 方圆 程, 小娥 王, 殿胜 钟, 琳琳 孙, 倩 王, 畅 刘

**Affiliations:** 1 300052 天津，天津医科大学总医院肿瘤科 Department of Medical Oncology, Tianjin Medical University General Hospital, Tianjin 300052, China; 2 300052 天津，天津肺癌研究所 Tianjin Lung Cancer Institute, Tianjin Medical University General Hospital, Tianjin 300052, China

**Keywords:** 肺肿瘤, 碳酸酐酶Ⅸ, 诊断, Lung neoplasms, Carbonic anhydrase Ⅸ, Diagnosis

## Abstract

**背景与目的:**

碳酸酐酶Ⅸ（carbonic anhydrase Ⅸ, CAIX）是一种跨膜蛋白，参与肿瘤细胞的代谢过程。其在少数正常组织中低表达，但在多种恶性肿瘤组织中广泛表达。检测CAIX在肺癌患者血清中的含量，探讨其对肺癌的诊断价值，分析不同病理类型及TNM分期肺癌患者血清CAIX含量是否存在差异。

**方法:**

选取47例肺癌患者和31例健康体检者为研究对象，用酶联免疫吸附测定（enzyme linked immunosorbent assay, ELISA）法检测其血清CAIX含量，根据病理类型及TNM分期分组，比较各组血清CAIX差异；绘制血清CAIX诊断肺癌的受试者工作特征曲线（receiver operating characteristic curve, ROC）。

**结果:**

肺癌组较健康对照组血清CAIX含量明显增高（*P* < 0.001）；鳞癌和小细胞癌患者血清CAIX含量明显高于腺癌患者。Ⅰ期+Ⅱ期与Ⅲ期+Ⅳ期的肺癌患者血清CAIX含量比较，未发现两者间的差异有统计学意义；血清CAIX诊断肺癌的ROC曲线下面积为0.961，当血清中CAIX阈值为115.115 pg/mL时，敏感度和特异度分别为95.7%和90.3%。

**结论:**

用ELISA法检测患者血清CAIX有助于肺癌诊断，且具有较高的敏感性和特异性。

## Abstract (en)

**Background and objective:**

Carbonic anhydrase Ⅸ (CAIX) is a transmembrane protein involved in the metabolism of tumor cells. CAIX is expressed in only a few normal tissues but is overexpressed in various tumor types. The aim of this study is to detect the serum CAIX level of patients with lung cancer, evaluate the significance of CAIX detection in the diagnosis of lung cancer, and analyze the serum CAIX level among different pathological types and TNM stages of lung cancer.

**Methods:**

Forty-seven patients with lung cancer and 31 healthy subjects were selected to participate in this study. Serum CAIX level was examined through enzyme-linked immunosorbent assay (ELISA). The subjects were grouped according to histological type and TNM staging, and serum CAIX level was compared among the groups. With pathological diagnosis as the gold standard, receiver operating characteristic curve of the serum CAIX level was established for the diagnosis of lung cancer.

**Results:**

The CAIX serum level was significantly higher in patients with lung cancer than that in the healthy group (*P* < 0.001). The serum CAIX level in patients with squamous cell carcinoma and small cell carcinoma was also significantly higher than that in patients with adenocarcinoma. No statistically significant differences were observed in the serum CAIX level between Ⅰ+Ⅱ and Ⅲ+Ⅳ staging. The AUC of serum CAIX level was 0.961. At a threshold level of 115.115 pg/mL, sensitivity and specificity were 95.7% and 90.3%, respectively.

**Conclusion:**

Detection of the serum CAIX level through ELISA exhibits high sensitivity and specificity and is important for the diagnosis of lung cancer.

原发性支气管肺癌（primary bronchogenic carcinoma, PBC），简称肺癌，不论是发病率还是死亡率均居全球癌症首位，积极提高肺癌的诊治水平具有十分重要的意义。临床工作中影像学、病理学是肺癌诊断的重要依据，血清中的肿瘤标记物检测已成为肺癌诊断的重要参考指标。目前常用的肿瘤标志物，如癌胚抗原、细胞角蛋白片段19、神经原特异性烯醇化酶等^[1]^，敏感性或特异性尚不能满足临床工作需要，因此，积极探索寻找更有意义的肿瘤标记物是现代肿瘤学研究的重要课题之一。

碳酸酐酶Ⅸ（carbonic anhydrase Ⅸ, CAIX）为跨膜蛋白，属于碳酸酐酶家族中一员。CAIX在人体正常组织中低表达，但在肺癌等多种恶性肿瘤组织中高表达，且在肿瘤细胞的生长、浸润及转移中起重要作用。研究发现，CAIX的细胞膜外部分可被蛋白酶水解，形成可溶性碳酸酐酶Ⅸ（soluble form of CAIX, s-CAIX），在血液中可以检测到，可用于肿瘤的诊断和治疗后评估。目前关于肺癌血清中CAIX的研究报道极少，本研究旨在探索血清CAIX对肺癌的诊断价值。

## 1 资料和方法

### 1.1 研究对象

#### 1.1.1 实验组

选自2012年6月-2013年3月在我院呼吸科、肿瘤科及肺外科住院治疗确诊为肺癌的47例患者，包括男性26例，女性21例。根据患者病理类型不同进行分类，包括鳞癌（*n*=7）、腺癌（*n*=26）、小细胞癌（*n*=10）及病理类型未明确（*n*=4）；按照肺癌TNM分期标准，包括Ⅰ期+Ⅱ期患者6例，Ⅲ期+Ⅳ期患者41例。实验组纳入标准：①气管镜活检组织和组织刷片、开胸肺活检组织、胸水液基及沉淀物经病理检查见肿瘤细胞，结合免疫组化染色及影像学检查最终诊断为肺癌；②入组前未接受放疗、化疗等任何抗肿瘤治疗。

#### 1.1.2 对照组

选自2012年7月-2012年12月在天津医科大学总医院体检中心体检的31例患者，包括男性18例，女性13例。对照组纳入标准：查体、化验及影像学等检查结果均无异常的健康体检者。

### 1.2 研究方法

#### 1.2.1 标本的采集和处理

所有研究对象于肘静脉抽取约3 mL静脉血，置于红盖非抗凝普通管，在4 ℃条件下以3, 000 r/min离心10 min，取出上清置于-40 ℃冰箱储存。于实验室检测前常温下一次性解冻所有标本。

#### 1.2.2 主要试剂

CAIX测定试剂盒购自武汉华美生物工程有限公司。

#### 1.2.3 检测方法

采用酶联免疫吸附测定（enzyme linked immunosorbent assay, ELISA）法检测患者血清中CAIX含量，具体操作严格按照说明书执行。

### 1.3 统计学方法

应用SPSS 17.0统计软件分析处理，对实验组和对照组数据进行正态性检验，因不符合正态分布，两组间比较采用两独立样本秩和检验，多组间比较采用多组独立样本的秩和检验，以*P* < 0.05表示差异具有统计学意义。绘制CAIX诊断肺癌的受试者工作特征曲线（receiver operating characteristic curve, ROC曲线）并计算曲线下面积（area under the curve, AUC）及曲线各坐标的敏感度和特异度。

## 2 结果

### 2.1 实验组及对照组患者血清中CAIX含量

实验组47例[男性26例，女性21例，年龄（65±11）岁]和对照组31例[男性18例，女性13例，年龄（61±10）岁]，两组间年龄差异（*t*=1.572, *P* > 0.05）及性别差异（*χ*^2^=0.57, *P* > 0.05）均无统计学意义。分别对两组实验数据进行正态性检验，两组结果均不符合正态分布（*P* < 0.05），故计量资料采用中位数和四分位数间距Md（Q）表示，两组间采用两独立样本秩和检验进行比较，结果显示实验组患者血清中CAIX含量为290.12（215.53-550.24）pg/mL，对照组血清中CAIX含量为56.54（41.83-94.18）pg/mL，实验组较对照组血清CAIX含量明显增高，且差异有统计学意义（*Z*=-6.876, *P* < 0.001）（图 1）。

**1:**
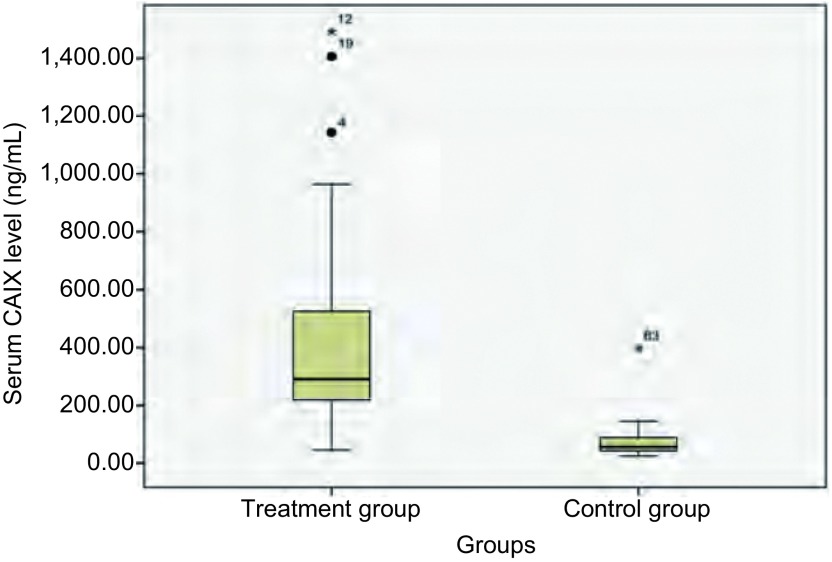
实验组与对照组患者血清中CAIX含量比较（pg/mL）。图中实验组4和19号标本CAIX含量超过1.5倍四分位数间距的值，为异常值，用“●”标出。实验组12号和对照组63号标本CAIX含量超出3倍四分位数间距的值，为极端值，用“*”标出。 Comparison of the serum CAIX level in the experimental group with the control group (pg/mL). The serum CAIX level of number 4 and number 19 in the treatment group was more than 1.5 times higher than the quartile range value, and was considered as the abnormal value and marked with "●". The serum CAIX level of number 12 in the treatment group and number 63 in the control group was more than 3 times higher than the quartile range value, and was considered as the extreme value and marked with "*". CAIX: carbonic anhydrase Ⅸ.

### 2.2 实验组中不同病理类型肺癌患者血清中CAIX含量比较

实验组47例患者根据病理类型不同分为鳞癌组（*n*=7）、腺癌组（*n*=26）、小细胞癌组（*n*=10），其余4例患者因病理类型不明确不参与统计分析。

分别对三组数据进行正态性检验，提示三组均不符合正态分布，采用中位数和四分位间距表示Md（Q），结果显示鳞癌组、腺癌组、小细胞肺癌组患者血清中CAIX含量分别为550.24（271.72-1, 142.93）pg/mL、257.39（202.39-404.32）pg/mL、529.36（241.49-933.34）pg/mL。采用多组独立样本的秩和检验分析三组实验数据，结果显示三组患者血清中CAIX含量差异有统计学意义（*χ*^2^=8.122, *P*=0.017）。进一步用两独立样本的秩和检验，分别对三组实验数据进行两两比较，结果显示：鳞癌组和小细胞癌组血清CAIX含量均高于腺癌组，差异均有统计学意义（*P* < 0.05），鳞癌组与小细胞癌组血清CAIX含量比较未发现两者间差异有统计学意义（*P* > 0.05）（表 1）。

**1:** 不同病理类型肺癌患者血清CAIX含量比较（pg/mL） Comparison of the serum CAIX level among different pathological types (pg/mL)

Control group	*Z*	*P*
SQC *vs* ADC ADC *vs* SCLC SQC *vs* SCLC	-2.335 -2.155 -0.195	0.018 0.031 0.845
SQC: squamous carinoma; ADC: adenocarcinoma; SCLC: small cell lung cancer.		

### 2.3 血清实验组Ⅰ期+Ⅱ期与Ⅲ期+Ⅳ期肺癌患者血清中CAIX含量

根据国际抗癌联盟（Union for International Cancer Control, UICC）2009年制定的第7版国际TNM分期标准，将47例肺癌患者分为Ⅰ期+Ⅱ期组（*n*=6）和Ⅲ期+Ⅳ期组（*n*=41）。

两组实验数据采用两独立样本的秩和检验，结果显示Ⅰ期+Ⅱ期肺癌患者血清中CAIX含量为330.10（250.97-754.65）pg/mL，Ⅲ期+Ⅳ期肺癌患者血清中CAIX含量为290.12（205.17-525.82）pg/mL，比较两者血清中CAIX含量，未发现两者间差异有统计学意义（*Z*=-0.781, *P*=0.444）。

### 2.4 绘制血清CAIX诊断肺癌的ROC曲线

以病理诊断为金标准，绘制血清CAIX诊断肺癌的ROC曲线，计算曲线下面积及各阈值的敏感度、特异度。血清CAIX的曲线下面积为0.961，血清中CAIX对于诊断肺癌有统计学意义（*P* < 0.05）。面积的95%CI: 0.914-1.008，不包括0.5，得出上述相同结论。当血清中CAIX阈值为115.115 pg/mL时，敏感度和特异度分别为95.7%和90.3%（图 2）。

**2:**
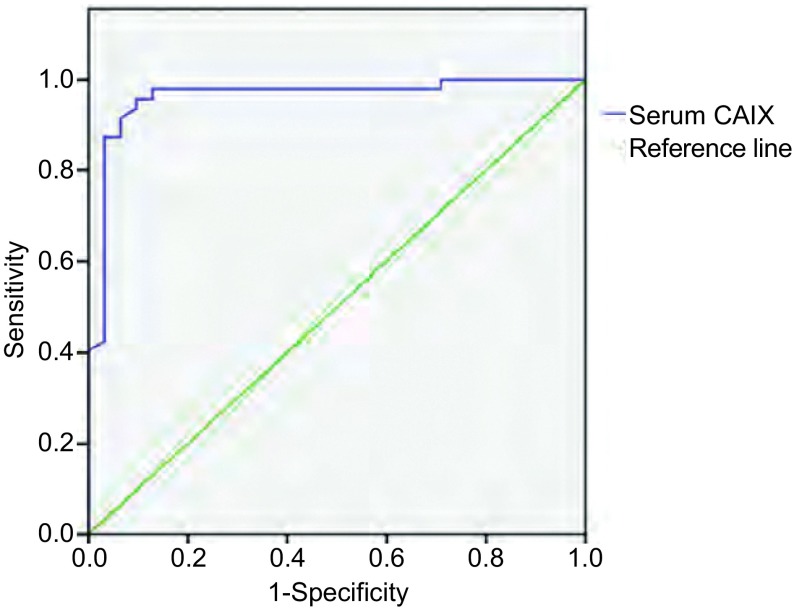
血清CAIX诊断肺癌的ROC曲线 The ROC curve of serum CAIX in the diagnosis of lung cancer. ROC: receiver operating characteristic curve.

## 3 讨论

CAIX是一种含锌金属蛋白酶，属于碳酸酐酶家族中一员，是一种新型的肿瘤抗原^[2]^。其相对分子质量为58 kDa或54 kDa^[3, 4]^，分为N末端信号肽、细胞外区、跨膜区以及细胞内C末端4个不同部分^[5]^。在低氧^[6]^、抑癌基因VLH（von Hippel-Lindau）突变或甲基化^[7, 8]^、细胞高密度^[9]^、表皮生长因子受体（epidermal growth factor receptor, EGFR）活化^[10]^等情况下可诱导CAIX表达增高。CAIX的生理作用是通过催化H^+^和HCO_3_^-^的可逆反应生成H_2_O和CO_2_，调节细胞内外酸碱度^[11]^。在活体肿瘤细胞内CAIX可促进H^+^与HCO_3_^-^转化为H_2_O和CO_2_，从而维持细胞内弱碱性环境，利于细胞的生长繁殖^[12]^。同时CAIX将肿瘤细胞外CO_2_与H_2_O转换为H^+^和HCO_3_^-^，HCO_3_^-^被细胞膜上的Cl^-^/ HCO_3_^-^泵泵入细胞内，而H^+^可降低细胞外PH值，酸性环境有利于肿瘤细胞对放化疗的抵抗^[13]^。因此，CAIX对肿瘤细胞生存及发展起重要调节作用。CAIX还可通过B-连环蛋白相互作用减少钙粘蛋白介导的粘附，利于肿瘤细胞浸润、转移^[14]^。

CAIX在胃粘膜细胞、胆囊的上皮细胞、胰腺胰管、小肠的隐窝细胞等少量正常组织中低表达^[15]^，但在多种恶性肿瘤组织中广泛表达，如在肺癌^[16, 17]^、肾癌^[18]^、宫颈癌^[19]^、外阴癌^[20]^、乳腺癌^[21]^等。Ivanov等^[6]^在87种恶性细胞株中发现其中50种可检测到CAIX mRNA，因此，CAIX可被视为广泛表达的肿瘤特异性标记物。

研究^[22, 23]^发现，支气管和肺泡中均无CAIX表达。通过对175例手术切除的非小细胞肺癌（non-small cell lung cancer, NSCLC）组织标本进行免疫组化染色，Swinson等^[8, 9]^发现CAIX表达阳性率达81.8%。Kim等^[24]^对75例手术切除的NSCLC标本进行免疫组化染色，发现CAIX在72%标本中表达，Ilie等^[16]^在一项较大研究中发现在24.3%（135/555）NSCLC的肿瘤组织中存在CAIX高表达。多项研究^[8, 22, 24, 25]^表明，NSCLC组织中CAIX的高表达与病理类型明显相关，鳞癌CAIX表达阳性率较腺癌高。Kon-No等^[26]^研究显示，CAIX表达阳性的患者无病生存期（disease-free survival, DFS）和总生存期（overall survival, OS）明显短于CAIX表达阴性的患者。

Zavada等^[3]^证实，CAIX细胞外的部分可被蛋白酶水解，生成s-CAIX，并释放到患者的血液、尿液中。Ilie等^[16]^运用ELISA法对209例术后经病理诊断明确的NSCLC患者血清中CAIX含量进行检测，结果显示，NSCLC血清中CAIX含量均值为45.40（0-372.89）pg/mL明显高于健康对照组均值2.48（0-16.65）pg/mL，两者差异有统计学意义。在本研究中，肺癌患者血清CAIX含量为290.12（215.53-550.24）pg/mL，正常对照组血清中CAIX含量为56.54（41.83-94.18）pg/mL，肺癌组明显高于对照组，差异有统计学意义，与Ilie等^[16]^结论一致。但本研究检测值明显高于文献中的检测值，考虑差异的产生与研究对象不同、种族差异、实验方法、使用的试剂盒及其灵敏度不同等有关。

文献报道肺鳞癌组织中CAIX阳性率高于腺癌，CAIX的表达与肿瘤组织坏死相关，而鳞癌较腺癌更易出现坏死。本研究也显示，肺鳞癌组血清CAIX含量高于腺癌组。目前尚未检索到小细胞癌患者血清CAIX含量的报道，本研究结果表明，小细胞肺癌患者血清CAIX含量较腺癌患者明显增高，差异具有统计学意义，但与鳞癌无差异。此外，血清CAIX诊断肺癌的ROC曲线研究显示，曲线下面积为0.961，提示血清中CAIX含量检测对于肺癌具有较高诊断价值。且当血清中CAIX阈值为115.115 pg/mL时敏感度和特异度较高，分别为95.7%和90.3%。

综上所述，血清中CAIX含量对于肺癌的诊断及病理类型的鉴别等具有一定的价值，在未来的临床工作中具有应用前景。
